# Gene functional similarity search tool (GFSST)

**DOI:** 10.1186/1471-2105-7-135

**Published:** 2006-03-14

**Authors:** Peisen Zhang, Jinghui Zhang, Huitao Sheng, James J Russo, Brian Osborne, Kenneth Buetow

**Affiliations:** 1 Laboratory of Population Genetics, National Cancer Institute, NIH, Bethesda, USA; 2 Columbia Genome Center, Columbia University, New York, USA; 3 Cognia Corporation, New York, USA

## Abstract

**Background:**

With the completion of the genome sequences of human, mouse, and other species and the advent of high throughput functional genomic research technologies such as biomicroarray chips, more and more genes and their products have been discovered and their functions have begun to be understood. Increasing amounts of data about genes, gene products and their functions have been stored in databases. To facilitate selection of candidate genes for gene-disease research, genetic association studies, biomarker and drug target selection, and animal models of human diseases, it is essential to have search engines that can retrieve genes by their functions from proteome databases. In recent years, the development of Gene Ontology (GO) has established structured, controlled vocabularies describing gene functions, which makes it possible to develop novel tools to search genes by functional similarity.

**Results:**

By using a statistical model to measure the functional similarity of genes based on the Gene Ontology directed acyclic graph, we developed a novel Gene Functional Similarity Search Tool (GFSST) to identify genes with related functions from annotated proteome databases. This search engine lets users design their search targets by gene functions.

**Conclusion:**

An implementation of GFSST which works on the UniProt (Universal Protein Resource) for the human and mouse proteomes is available at GFSST Web Server. GFSST provides functions not only for similar gene retrieval but also for gene search by one or more GO terms. This represents a powerful new approach for selecting similar genes and gene products from proteome databases according to their functions.

## Background

Cellular function in a biological system normally involves participation and interaction of multiple genes. Mutations that alter function of any one of these genes can potentially increase disease susceptibility. For example, the tumor suppressor gene BRCA1 suppresses cell growth and participates in transcription-coupled DNA damage repair. Mutations in BRCA1 increase the risk of early onset breast cancer as well as ovarian and prostate cancer [[1,2], and [3]]. Genes with functions similar to BRCA1 can be considered additional candidate genetic risk factors for breast, ovarian, prostate, or other cancers.

One common approach to identify functionally similar genes is to find genes that share significant sequence homology. However, functional similarity does not always require sequence similarity. For example, both P53 [4] and BRCA1 [5] function as tumor suppressor genes. Similar to BRCA1, mutations in P53 have also been found in breast cancer patients [6]. The two genes share no sequence homology. As a result, a sequence similarity search tool, such as BLAST [7], is unable to reveal their functional similarity.

An alternative to sequence homology search is key word search, but this approach has two weaknesses. First, key words for gene functions are not well defined. Second, key word search is a static binary operation and therefore cannot quantify the significance of the search results, which makes it difficult for a user to evaluate the outcome.

In recent years, the GO Consortium has made major advances in establishing structured, controlled vocabularies describing gene functions. Each vocabulary is structured as a directed acyclic graph (DAG), wherein any term may have more than one parent term as well as zero, one, or more child terms. The DAG structure provides a much richer description of the underlying biology than would be possible with a strictly hierarchical tree graph. GO describes genes and proteins using three categories: Biological Process, Molecular Function, and Cellular Component [[8] and [9]]. "Molecular Function (MF) describes catalytic or binding activities, at the molecular level. Individual molecular function terms include the broad concept such as 'kinase activity' and the more specific '6-phosphofructokinase activity', a subtype of kinase activity. Biological Process (BP) describes biological processes accomplished by one or more ordered assemblies of functions. High-level processes such as 'cell death' can have subprocesses, such as 'apoptosis' and 'apoptotic chromosome condensation'. Cellular Component (CC) describes subcellular structures and macromolecular complexes. Examples of cellular components include 'nuclear inner membrane', with the synonym 'inner envelope', and the 'ubiquitin ligase complex', with several subtypes of these complexes represented." Currently, the GO vocabulary consists of more than 18,000 terms [[10,11], and [12]].

UniProt (Universal Protein Resource) is a comprehensive catalogue of information on protein sequence and function created by integrating data from Swiss-Prot, TrEMBL, and PIR. UniProt is the central access point for extensive curated protein information, including function, classification, and cross-reference. GO assignments have been applied to data sets in UniProt representing the complete human and mouse proteomes by a combination of electronic mappings and manual curation [[10] and [11]]. This vocabulary has also been applied to the nonredundant proteome sets in UniProt for all other completely sequenced organisms as well as to proteins from a wide range of organisms where the genome is not yet complete.

Recently, some web servers (UCSC Gene Sorter [13] and GOToolBox [14]) have provided gene search engines for shared Gene Ontology (GO) terms and some stand-alone software packages (GO Graph [15] and DynGO [16]) also provide gene similarity search functions.

Using different similarity measures and search methodologies, we have developed a search engine: Gene Functional Similarity Search Tool (GFSST), to identify candidate genes based on their functional similarities. We have implemented GFSST for the human and mouse proteomes in the UniProt database. For a given protein with known functions or a set of protein functions, and a given proteome, the GFSST search engine not only can find any proteins in the proteome with the same functions (shared GO terms) but also can discover proteins with similar functions (not necessarily shared, but with very similar GO terms). We have defined a statistical measure: D-value (Distribution value) to quantify functional similarity of genes based on the GO directed acyclic graph (DAG). There are three levels of definition for D-values. First, the D-value for each GO term on GO DAG is defined as a positive value with additive and monotonic properties. The D-value for each term is the sum of the D-values of its direct children (additive property). Obviously, the D-value of the child cannot be greater than the D-value of its parent (monotonic property). In its current implementation, the D-value at the root of GO DAG is assigned a value of 1, and all leaf terms have the same D-value. Then, the D-value for every pair of GO terms is defined as the minimum D-value of their common parent terms. For identical GO terms, the D-value is set to zero. The D-value for a pair of genes is defined as the mean of the D-values for their matched GO term pairs. More details can be found in the METHODS section and in the DISCUSSION.

## Results

We have developed a GFSST search engine for human and mouse proteomes. Input data can be a set of GO terms or a protein identifier (either a protein name or an accession number) in the human or mouse UniProt database. The search can be carried out against the human or the mouse proteome. The search engine can also be used as retrieval tool for the human and mouse UniProt databases.

### Search by a source protein

**Figure 1:**
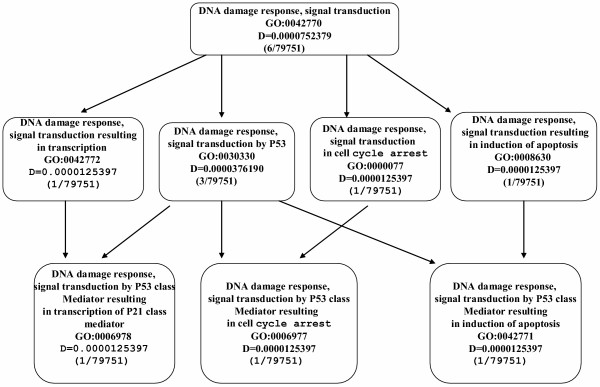
Subgraph of the GO Biological Process directed acyclic graph (data source: DAG-Edit Version 1.419 Rev 3). Each box represents a GO term with term definition, GO ID, and D-value D; there are two forms for D-value, decimal and fractional; the nominator in the fraction is the count of the GO term, the denominator is the count of the whole GO DAG root.

Given a source protein, GFSST can retrieve functionally related proteins from a specific proteome. To illustrate this process, we used human BRCA1 as an example. BRCA1_HUMAN (accession # P38398) in UniProt (EBI GOA_human release 28) has 9 GO terms in the Biological Process category (Table 1). Most of those terms refer to DNA repair and regulation of apoptosis. The top 10 hits of BRCA1_HUMAN searched by GFSST are listed in Table 2, one of which is P53_HUMAN. The two genes share one common GO term GO:0045786 (negative regulation of cell cycle) and 8 similarly matched pairs of GO terms. One of the pairs is GO:0006978 (DNA damage response, signal transduction by P53 class mediator resulting in transcription of P21 class mediator) and GO:0008630 (DNA damage response, signal transduction resulting in induction of apoptosis). Their common parent with minimum D-value 0.0000752370 is GO:0042770 (DNA damage response, signal transduction) (Figure 1). So the D-value for the pair of GO terms is 0.0000752370. More GO terms matched for P53 and BRCA1 are shown in Table 3. There are a total of 97 hits with D-values < 0.05 in the three GO categories after BRCA1_HUMAN search by GFSST, one of which is BRCA2_HUMAN with D-value 0.0476423351.

In mouse model research, it is important to find mouse proteins that are functionally similar to a human protein. The top 10 matches with D-values < 0.05 in searching the UniProt mouse proteome (EBI GOA_mouse release 14) with gene BRCA1_HUMAN in the Biological Process category are listed in Table 4.

### Search by GO terms

GFSST provides a robust retrieval tool for gene and gene products based on their associated GO terms. This is a more flexible approach than searching with a source protein. Users can design their search targets by a single or a combination of GO terms. Given GO terms, GFSST can retrieve gene products from a specific proteome. Thus users can design their search by providing a set of gene functions (GO terms). GFSST can find genes or gene products matched by those functions and/or by similar functions.

For example, glucose metabolism is a critical pathway in the study of diabetes. The target proteins with the glucose metabolism function (GO term GO: 0006006) in the Biological Process category will thus be relevant to diabetes. GFSST delivered 19 exact matches for this GO term in the UniProt human proteome. Insulin tops the search results.

DNA damage response, signal transduction by P53 class mediator (GO:0030330) is a very important function in cancer research. Performing GFSST search for this GO term in the Biological Process category we find no exactly matching gene products in the UniProt human proteome. There are four protein hits, including BRCA1_HUMAN matched by GO:0006978 (a child of GO:0030330) with D-value 0.0000376, and P53_HUMAN and P73_HUMAN matched by GO:0008630 with D-value 0.0000752370. It is not surprising that there are no exact matches for the term GO:0030330, because there are some more specific terms under GO:0030330, its children, that are assigned for gene products.

Users can also query a set of GO terms to obtain target proteins for the biological functions of interest. For example, angiogenesis inhibitors are designed to stop tumors from developing a blood supply, a prerequisite for tumor growth and metastasis. Four GO terms, GO:0016525 (negative regulation of angiogenesis), GO:0008285 (negative regulation of cell proliferation), GO:0042981 (regulation of apoptosis), and GO:0006917 (induction of apoptosis), describe the biological processes involved in angiogenesis inhibition. GFSST found 402 proteins with D-value < 0.05 via the above four GO terms. The first 10 proteins are listed in Table 5.

## Discussion

GFSST is a new search engine that facilitates the selection of candidate genes and gene products for human disease, for mouse models, for biomarkers, and for drug targets. GFSST can retrieve function-specific gene products (such as proteins, antibodies, and enzymes) from the UniProt database based on assigned ontology annotations rather than sequence, sequence motif, or other sequence attributes. The GFSST search quality will improve with the increasing accuracy and completeness of GO DAG and gene annotation by GO terms. Its usefulness will grow as biological studies expand our current knowledge of the proteome.

It is an important and unique feature of GFSST that users are able to retrieve genes not only from annotated genes, but also from a combination of GO terms. GFSST can retrieve not only the genes with shared GO terms but also the genes with very similar (not necessarily the same) GO terms. In order to make GFSST useful not only for users who are familiar with GO terms, more technologies may be needed such as natural language search tools.

Well-defined D-values for GO terms, for a pair of GO terms, and for a pair of annotated genes (equivalent to a pair of sets of GO terms) are the foundation of GFSST. We have not yet considered evidence codes in the calculation of D-values. For stronger evidence, a heavier weighting factor should be used. We plan to add evidence codes to our algorithm in the next version of our web server.

It is possible to assign different distributions to GO terms. Lord and his collaborators [15] have used the frequencies of the matches to GO terms by UniProt gene products as distributions. We can calculate probabilities *p *for GO terms and pairs of GO terms exactly as done in [15], and then calculate the D-value for two groups of GO terms with our greedy pairing algorithm. Using this D-value system, we have tested GFSST for BRCA1_HUMAN. Different but similar results are obtained. Outputs obtained using the two measures for BRCA1 gene similarity search are listed in Table 6 (data source: DAG-Edit version 1.416; EBI GOA_human release 19). Our D-value distributions do not need to calculate the hit frequencies, and are not sensitive to annotation changes; they are in fact essentially annotation-independent. In other words, the D-value is a universal measure for GO terms and pairs of GO terms, and so can be used for any GO annotated database search. The similarity measure used in [15] is dependent on the probability of the specific term occurring in the annotated database, and thus is not a universal measure. This becomes a significant issue for cross-species, such as human-to-mouse, searches, and searches against sources with different annotations, since separate probabilities, *p*(c), would need to be calculated for every individual and every pair of species/database similarity searches. Nonetheless, in future releases of GFSST, an option for selecting measures other than D-values will be provided for limited databases.

There are some available web servers for gene similarity search that are not entirely restricted to homology. The UCSC Gene Sorter [13] "displays a table of genes within a selected genome that are related to one another." The GOToolBox [14] "has developed methods and tools based on the Gene Ontology (GO) resource allowing the identification of statistically over- or under-represented terms in a gene dataset; the clustering of functionally related genes within a set; and the retrieval of genes sharing annotations with a query gene." We tested Gene Sorter and GOToolBox with the source protein BRCA1_HUMAN. A comparison of the results from GFSST, Gene Sorter and GOToolBox is presented in Table 7. Most of the GOToolBox gene products listed in Column 2 of Table 7 fall within the parts of the ontology dealing with apoptosis, androgen binding, and similar categories, perhaps because the algorithm will usually search for terms no more than one step up in the GO DAG. In contrast, the hits using GFSST (Column 1) seem to cover a broader spectrum of the ontology, *e.g*., DNA damage response, cell proliferation, centriole regulation, receptor signaling in addition to those already mentioned, due to the fact that the computation is not limited to only one step up the hierarchy. Hence, GFSST provides the opportunity to find unsuspected gene products that may cooperate (either directly or indirectly) to subserve larger biological, enzymatic or metabolic functions, for example shared elements of multiple signal transduction pathways. In the case of the GO search directed from the UCSC Browser (Column 3), only those gene products which have been characterized in the available gene expression studies, albeit quite a large portion of the known genes, will be targeted; this may explain the different hits, for a perhaps narrower range of specific categories, and a few seemingly more farfetched ones.

The similarity measures based on the shared GO terms are simple and useful methodologies. But they are limited to the fact that if there are no shared terms, the similarity score (similarity percentage formulas (3), (4), and (5) in [14]) will be 0 even if two genes have very similar functions. Recognizing this weakness, the authors of GOToolBox extended the shared GO term from "common associated term" to "common parent terms" [14]. We understand this to mean the most immediate "parent terms". Our D-value measurement defines a similarity score for every pair of GO terms. For example, the pair GO: 0006978 (DNA damage response, signal transduction by P53 class Mediator resulting in transcription of P21 class mediator) and GO: 0008630 (DNA damage response, signal transduction resulting in induction of apoptosis) do not have the most immediate one step up parent (see Figure 1). GOToolBox considered this pair not shared. In contrast, the D-value in GFSST is 0.0000752370 for this pair, which is one of the aligned pairs for BRCA1_HUMAN and P53_HUMAN (see Table 3). In Table 7, GFSST listed P53_HUMAN as the protein with the most significant score for the BRCA1_HUMAN search, but it is not at the top of the GOToolBox search results. It is very interesting that Gene Sorter listed TP53 at the top of its output, which may be explained by the fact that, in addition to shared terms, Gene Sorter uses popularity score in its search.

In short, different search engines have different algorithms and different features. If one wishes to find similar genes with shared GO terms, Gene Sorter and GOToolBox are good choices. If one is interested not only in shared, but also similar GO terms, GFSST is preferable. Beyond that, GFSST allows users the flexibility to design their search targets.

## Conclusion

An implementation of GFSST which works on the UniProt (Universal Protein Resource) for the human and mouse proteomes is available at GFSST Web Server [17]. GFSST provides functions not only for similar gene retrieval but also for gene search by one or more GO terms. This represents a powerful new approach for selecting similar genes and gene products from proteome databases according to their functions.

## Methods

### Statistical measures on the GO Directed Acyclic Graph (DAG)

Expressions in the GO are descriptions of concepts situated in an ontology formed by a DAG with concept inclusion as the ordering relation from less specialized terms (parents) to more specialized terms (children). In order to keep the ontology consistent in concept, there are no cycles in the DAG representation. We use a recursive procedure to define a D-value for each term in the DAG. First, we set the count to 1 for each leaf. Then we assign the count for every term recursively by summing the counts of all its direct children from the bottom up. For example, in Figure 1 (a subgraph of Biological Process GO DAG, DAG-Edit version 1.419 rev 3), there are three leaf terms GO:0006978, GO:0006977, and GO:0042771; each is assigned a count of 1. The term GO:0030330 is assigned a count of 3 by summing the counts of its direct children. The other terms at the same level as GO:0030330 have only one direct child each, and each direct child has count 1; each of these terms is thus assigned a count of 1. The term GO:0042770 therefore has direct children with counts 1, 3, 1 and 1, and is thus assigned a count of 6. According to previous reports [18], the distributions for terms on the GO DAG are monotonically nondecreasing, moving from child term to parent term. In our implementation, the distribution (D-value) for a term is its count divided by the count of the root of the whole GO DAG. As shown in Figure 1, the D-value equals the count divided by 79751 (the count for the root of the Biological Process GO DAG). Since the count for any term is the sum of the counts of its direct children, the distribution of a term is the sum of the distributions of its direct children. In addition to its monotonic property, our distribution is additive.

We define the D-value for a pair of GO terms based on the definition of D-value for a single GO term. For a pair of identical terms, it is natural to define the pair's D-value as equal to 0. Given two different terms c1 and c2 on the GO DAG, they can share parents by multiple paths, as GO allows multiple parents for each term. Let P (c1, c2) denote the set of parental terms shared by both c1 and c2. We define the D-value for the pair of terms c1 and c2 as the minimum D-value of their parents, as shown in the following equation.

D(c1,c2)=min⁡c∈P(c1,c2){D(c)}
 MathType@MTEF@5@5@+=feaafiart1ev1aaatCvAUfKttLearuWrP9MDH5MBPbIqV92AaeXatLxBI9gBaebbnrfifHhDYfgasaacH8akY=wiFfYdH8Gipec8Eeeu0xXdbba9frFj0=OqFfea0dXdd9vqai=hGuQ8kuc9pgc9s8qqaq=dirpe0xb9q8qiLsFr0=vr0=vr0dc8meaabaqaciaacaGaaeqabaqabeGadaaakeaaieGacqWFebarcqGGOaakcqqGJbWycqaIXaqmcqGGSaalcqqGJbWycqaIYaGmcqGGPaqkcqGH9aqpdaWfqaqaaiGbc2gaTjabcMgaPjabc6gaUbWcbaGaee4yamMaeyicI4SaeeiuaaLaeiikaGIaee4yamMaeGymaeJaeiilaWIaee4yamMaeGOmaiJaeiykaKcabeaakiabcUha7jab=reaejabcIcaOiabbogaJjabcMcaPiabc2ha9baa@4C4F@

The above formula to define the D-value for a pair of GO terms is the same as formula (1) in [15] to define the *probability of the minimum subsumer*. Instead of using distribution D(c) for GO terms, Lord *et al *use a frequency probability, *p*(*c*). Both definitions are monotonic.

We implement the GFSST application in four steps in the preparation phase. First, we construct an objective class for Gene Ontology structure: the specific directed acyclic graph. Second, a topological search is performed for the GO DAG. Any DAG can be ordered along a vertical line so that all directed edges go from up to down and all children are under their parents [19]. Third, we set the count to 1 for leaf terms. Then we calculate the counts for non-leaf terms by recursive addition on topologically ordered terms from bottom to top, and calculate the D-value by dividing counts by the total count. An example of D-value calculation is shown in Figure 1. First, the counts are calculated, for example, the GO:0042770 has 6 counts and its distribution is 0.0000752379 (6 divided by 79751, the counts on the root). In the last step, D-values for all pairs of GO terms are generated by choosing the minimum D-values of their parent GO terms. In Figure 1, leaf terms GO:0006978 and GO:0042771 have two common parents: GO:0042770 and GO:0030330. GO:0030330 has a smaller D-value than GO:0042770 (0.000037619 vs. 0.0000752379). The D-value of the pair will thus be 0.0000376190.

In order to understand the meaning of the D-value from a biological point of view, we provide some examples. The pair GO:0006978 and GO:0042771 describe very similar functions. Both belong to the function subcategory DNA damage response, signal transduction by P53 (GO:0030330). GO:0006978 is specific to mediator resulting in transcription of P21 class mediator and GO:0042771 is specific to mediator resulting in induction of apoptosis. The D-value of the pair is very small at 0.0000376. But the pair GO:0006978 and GO:0009631 (cold acclimation) has D-value 0.004138; their common parent GO term with minimum D-value is GO:0006950 (response to stress). They are very different biological functions than the pair GO:0006978 and GO:0042771.

### Statistical measures for gene products

Gene products (proteins) often have more than one term in the GO DAG. In order to retrieve all genes associated with the same and/or similar terms from a given proteome, it is necessary to develop a measure between two term groups on the GO DAG. Instead of using a simple average formula [[15] &[16]] or the Czekanowski-Dice formula [14], we have formulated an algorithm by paring the GO terms first then averaging the D-values for the matched pairs of GO terms. There are three cases to consider in the greedy pairing phase.

(1) For a pair of gene products with the same number (*N*) of terms on GO DAG, every term for one gene product is matched *once and only once *to a term for another gene product. There are *N *factorial numbers of combinations. The best solution will be one with the minimum sum of their D-values, but determining the minimum is an NP-complete problem. We have developed a greedy algorithm to obtain an approximate solution. First we calculate D-values for all pairs of terms from different gene products. After sorting all pairs according to D-value, we choose pairs one by one, starting from the pair with the minimum D-value, following the *once and only once *rule.

(2) If the source gene product has fewer terms than the target gene product, we choose the same number of terms from the target gene product so that the sum of the D-values generated by our greedy algorithm is the minimum. In our implementation, the same greedy procedure is performed, since the number of terms from the source gene product is less than the number of terms from the target gene product. When the *once and only once *rule is satisfied for the source gene product, the solution is obtained and the procedure is finished. Thus some terms from the target gene product will have no matches.

(3) If the source gene product has more terms than the target gene product, the *once and only once *rule should be followed for source gene product and the *at least once rule *is followed for target gene product. Thus some terms from the target gene product will be matched more than once.

In summary, there are two rules that are followed in our algorithm: the minimization of the final D-value and the *once and only once *rule for the source gene product. After pairing, it is straightforward to get the average.

In order to understand the meaning of the D-value for two genes or gene products, we present some examples. We have calculated the D-values for two pairs of genes in the Biological Process category. The pair BRCA1_HUMAN and P53_HUMAN has D-value 0.0064676759; and the pair BRCA1_HUMAN and SIAS_HUMAN (Sialic acid synthase) has D-value 0.1631506857. From a biological point of view, P53 is closer to BRCA1 than SIAS, consistent with our D-values.

Our algorithm is different from a simple average [15,16]. In the simple average formula, every GO term in one group will match every GO term in the other group, which does not always make sense. For two identical groups of GO terms or identical genes, it makes sense to match only identical GO terms. Our algorithm guarantees identical matches. Therefore the average D-value is obviously 0, the best value.

The Czekanowski-Dice [14] "formula emphasizes the importance of the shared GO terms by giving more weight to similarities than to differences. Consequently, for two genes that do not share any GO terms, the distance value is 1, the highest possible value, whereas for two genes sharing exactly the same set of GO terms, the distance value is 0, the lowest possible value."

GFSST emphasizes the importance of the shared GO terms in a different way. First every hit should reach the threshold D-value. Then GFSST presents the search results by sorting according to the number of exact matches. If there are no shared GO terms, search engines based on "shared GO terms" will return nothing, but GFSST still can retrieve similar gene products (see the GO:0030330 search results in the RESULTS section).

## Authors' contributions

PZ designed and implemented the search engine. JZ designed the distribution measure for DAG. HS designed and implemented the web interface. KB, JZ, JR, and BO participated in the overall design.

## Figures and Tables

**Table 1:** Functions of the BRCA1_HUMAN protein in GO Biological Process category (data source: EBI goa_human.28)

**GO Term ID**	**Definition of the GO Term**
GO:0042981	regulation of apoptosis
GO:0045786	negative regulation of cell cycle
GO:0006978	DNA damage response, signal transduction by p53 class mediator resulting in transcription of p21 class mediator
GO:0046600	negative regulation of centriole replication
GO0016567	protein ubiquitination
GO:0045739	positive regulation of DNA repair
GO:0042127	regulation of cell proliferation
GO:0006357	regulation of transcription from Pol II promoter
GO:0006359	regulation of transcription from RNA polymerase III promoter

**Table 2:** The top 10 matches to the BRC1_HUMAN gene after searching the UniProt human proteome with a D-value cutoff of 0.05 in the Biological Process Category (data source: DAG-Edit version 1.419 Rev 3 and EBI goa_human.28)

**Protein**	**Summary of functions**	**D-value**
BRCA1_HUMAN	Plays a central role in DNA repair by facilitating cellular response to DNA repair	0.000000000
P53_HUMAN	Acts as a tumor suppressor in many tumor types	0.0064676759
VHL_HUMAN	Von Hippel-Lindau disease tumor suppressor	0.0094604740
P73_HUMAN	Participates in the apoptotic response to DNA damage	0.0102616186
INHA_HUMAN	Inhibin alpha chain [Precursor]	0.0119586522
INHBA_HUMAN	Inhibin beta A chain [Precursor]	0.0119586522
CGRF1_HUMAN	Cell growth regulator with RING finger domain 1	0.0120311036
MAT1_HUMAN	CDK-activating kinase assembly factor MAT1	0.0189669271
PMS1_HUMAN	Probably involved in the repair of mismatches in DNA	0.0191020767
RBBP8_HUMAN	Retinoblastoma-binding protein 8	0.0191020767
IRF1_HUMAN	Specifically binds to the upstream regulatory region of type I IFN and IFN-inducible MHC class I genes (the interferon consensus sequence (ICS)) and activates those genes.	0.0238448534

**Table 3:** GO terms that match P53_HUMAN and BRCA1_HUMAN in the Biological Process category

**BRCA1_HUMAN**	**P53_HUMAN**	**D-value**	**Common Parent**
GO:0045786	GO:0045786	0.0000000000	Exact Match
GO:0006978	GO:0008630	0.0000752370	GO:0042770
GO:0042981	GO:0008628	0.0003009510	GO:0042981
GO:0045739	GO0006284	0.0007899980	GO:0006281
GO:0042127	GO0008283	0.0011285690	GO:0050875
GO:0006357	GO:0006355	0.0012163460	GO:0006355
GO:0046600	GO0046902	0.0037869760	GO:0006996
GO:0016567	GO0051262	0.0221701130	GO:0044267
GO:0006359	GO0006289	0.0287408930	GO:0006139

**Table 4:** The top 10 matches to BRCA1_HUMAN from the UniProt mouse proteome obtained by searching the Biological Process category with a D-value cutoff of 0.05 (data source: DAG-Edit version 1.419 Rev 3, and EBI goa_mouse.14)

**Protein**	**Summary of functions or protein name**
BRCA1_MOUSE	Plays a central role in DNA repair by facilitating cellular response to DNA repair
SMRA3_MOUSE	SWI/SNF-related matrix-associated actin-dependent regulator of chromatin subfamily A member 3
BCL10_MOUSE	Promotes apoptosis, pro-caspase-9 maturation and activation of NF-kappa-B via NIK and IKK.
INHA_MOUSE	Inhibins and activins inhibit and activate, respectively, the secretion of follitropin by the pituitary gland.
DDIT3_MOUSE	Growth arrest and DNA-damage-inducible protein GADD153
ATM_MOUSE	Involved in signal transduction, cell cycle control and DNA repair. May function as a tumor suppressor. Necessary for activation of ABL1 and SAPK
ASC_MOUSE	Promotes caspase-mediated apoptosis.
HMG2_MOUSE	High mobility group protein 2
CAF1A_MOUSE	Complex that is thought to mediate chromatin assembly in DNA replication and DNA repair.
MLH1_MOUSE	DNA mismatch repair protein Mlh1

**Table 5:** The first 10 matches with D-value less than 0.05 in the human proteome database for a set of GO terms (GO:0006917, GO:0008285, GO:0042981, and GO:0016525).

**Protein ID**	**Protein Name**	**D-value**
CO4A3_HUMAN	Collagen alpha 3(IV) chain [Precursor]	0.0001473407
Q9NYC4	Tumstatin [Fragment]	0.0004294830
BTG1_HUMAN	B-cell translocation gene 1 protein	0.0016740440
NOTC2_HUMAN	Neurogenic locus notch homolog protein 2 [Precursor]	0.0027179702
LX15B_HUMAN	Arachidonate 15-lipoxygenase, type II	0.0043105068
DEDD_HUMAN	Death effector domain-containing protein	0.0170946868
RTN4_HUMAN	Reticulon-4, Neurite outgrowth inhibitor	0.0029342793
TNR7_HUMAN	Tumor necrosis factor receptor superfamily member 7 [Precursor]	0.0030001125
APAF_HUMAN	Apoptotic protease-activating factor 1	0.0044578475
NUPR1_HUMAN	Nuclear protein 1	0.0051569335

**Table 6:** Comparison of the search results (the first 10 outputs) obtained using the simple uniform (D-value) distribution (Column 1) and the distribution based on the matches to UniProt entries (Column 2) for gene BRCA1_HUMANin the Biological Process category (data source: DAG-Edit version 1.416; EBI GOA_human release 19)

**Simple**	**UniProt Matches**
BRCA1_HUMAN	BRCA1_HUMAN
P73_HUMAN	P73_HUMAN
PMS1_HUMAN	PMS1_HUMAN
P53_HUMAN	IRF1_HUMAN
IRF1_HUMAN	P53_HUMAN
FOS_HUMAN	TFH1_HUMAN
Z161_HUMAN	CYCH_HUMAN
MLH1_HUMAN	FXN3_HUMAN
MSH2_HUMAN	RFC1_HUMAN
PMS2_HUMAN	AHR_HUMAN

**Table 7:** Comparison of the search results (the first 11 outputs) for gene BRCA1_HUMAN for GFSST, GOToolBox (both in the Biological Process category) and UCSC Gene Sorter by choosing GO Similarity

**GFSST**	**GOToolBox**	**Gene Sorter**
BRCA1_HUMAN	BRCA1_HUMAN	BRCA1
P53_HUMAN	CDK7_HUMAN	TP53
VHL_HUMAN	RNF4_HUMAN	BARD1
P73_HUMAN	RNF14_HUMAN	BIRC2
INHA_HUMAN	Q96PK6	BIRC3
INHBA_HUMAN	PIAS2_HUMAN	MNAT1
CGRF1_HUMAN	TIP60_HUMAN	PML
MAT1_HUMAN	PIAS1_HUMAN	RAD18
PMS1_HUMAN	ABL1_HUMAN	RNF14
RBBP8_HUMAN	P73_HUMAN	TIF1
IRF1_HUMAN	VHL_HUMAN	TRIM28
